# Impact of Cold versus Hot Brewing on the Phenolic Profile and Antioxidant Capacity of Rooibos (*Aspalathus linearis*) Herbal Tea

**DOI:** 10.3390/antiox8100499

**Published:** 2019-10-21

**Authors:** Elisabetta Damiani, Patricia Carloni, Gabriele Rocchetti, Biancamaria Senizza, Luca Tiano, Elizabeth Joubert, Dalene de Beer, Luigi Lucini

**Affiliations:** 1 Department of Life and Environmental Sciences, Università Politecnica delle Marche, Via Brecce Bianche, I-60131 Ancona, Italy; e.damiani@univpm.it (E.D.); l.tiano@univpm.it (L.T.); 2 Department of Agricultural, Food and Environmental Sciences, Università Politecnica delle Marche, Via Brecce Bianche, I-60131 Ancona, Italy; p.carloni@univpm.it; 3 Department for Sustainable Food Process, Università Cattolica del Sacro Cuore, Via Emilia Parmense 84, 29122 Piacenza, Italy; biancam.senizza@virgilio.it (B.S.); luigi.lucini@unicatt.it (L.L.); 4 Plant Bioactives Group, Post-Harvest & Agro-Processing Technologies, Agricultural Research Council, Infruitec-Nietvoorbij, Private Bag X5026, Stellenbosch 7599, South Africa; JoubertL@arc.agric.za (E.J.); dbeerd@arc.agric.za (D.d.B.); 5 Department of Food Science, Stellenbosch University, Private Bag X1, Matieland (Stellenbosch) 7602, South Africa

**Keywords:** rooibos tea, metabolomics, polyphenols, in vitro antioxidant capacity, cold brewing, hot brewing

## Abstract

Consumption of rooibos (*Aspalathus linearis*) as herbal tea is growing in popularity worldwide and its health-promoting attributes are mainly ascribed to its phenolic composition, which may be affected by the brewing conditions used. An aspect so far overlooked is the impact of cold brewing vs regular brewing and microwave boiling on the (poly) phenolic profile and in vitro antioxidant capacity of infusions prepared from red (‘fermented’, oxidized) and green (‘unfermented’, unoxidized) rooibos, the purpose of the present study. By using an untargeted metabolomics-based approach (UHPLC-QTOF mass spectrometry), 187 phenolic compounds were putatively annotated in both rooibos types, with flavonoids, tyrosols, and phenolic acids the most represented type of phenolic classes. Multivariate statistics (OPLS-DA) highlighted the phenolic classes most affected by the brewing conditions. Similar antioxidant capacities (ORAC and ABTS assays) were observed between cold- and regular-brewed green rooibos and boiled-brewed red rooibos. However, boiling green and red rooibos delivered infusions with the highest antioxidant capacities and total polyphenol content. The polyphenol content strongly correlated with the in vitro antioxidant capacities, especially for flavonoids and phenolic acids. These results contribute to a better understanding of the impact of the preparation method on the potential health benefits of rooibos tea.

## 1. Introduction

In recent years, the herbal tea prepared from stems and leaves of the endemic South African leguminous shrub—*Aspalathus linearis*, commonly known as rooibos or ‘Red Bush’ tea, has carved a niche for itself as a refreshing, caffeine-free, healthy drink, enjoyed by consumers of all ages from children to the elderly, and even by pregnant women and their new-born babies. A summary of in vitro and in vivo studies on the bioactivity of rooibos infusions or extracts can be found in [1]. Rooibos is characterized by the presence of aspalathin (a dihydrochalcone), which has been found to be a major compound contributing to the antidiabetic properties of this herbal tea [2,3]. This aspect makes rooibos extracts interesting as a condition-specific antioxidant, as such antioxidants that deliver more than just the ambiguous benefit of antioxidant activity, are in demand by the nutraceutical industry [4]. The levels of this unique antioxidant vary between the traditional ‘fermented’ (oxidized) rooibos which is red-brown in color, and the less popular variant of ‘unfermented’ (unoxidized) rooibos which is green. It is in fact well known that during the natural ‘fermentation’ process, the aspalathin content substantially decreases due to oxidative conversion to flavanones and flavones, amongst others, which also affects the total polyphenol content [5,6]. However, the fermented tea is characterized by a slightly sweet taste and flavor with honey, woody and herbal-floral notes [7], unlike the unfermented version which has a predominant grassy aroma and flavor.

Several studies have also shown how the antioxidant capacity and polyphenol content of rooibos are affected by different brewing times (1–10 min), temperatures (65–100 °C), household flavoring additions (sugar, milk, honey) and type (loose or bagged plant material or powdered extracts) [8,9,10,11,12]. This aspect is particularly important considering that rooibos is becoming extremely popular in the Far East, especially in Japan, one of the largest tea-drinking nations in the world. Exports of rooibos to this country hit record highs in 2018 [13]. In Japan, rooibos tea is commonly prepared both as a hot and cold brew. Cold brewing implies infusing the plant material in water at room temperature (or cold) and not in boiling water, followed by chilling (as is generally the case for preparation of iced tea). We previously studied hot and cold infusions of black, green and white tea (*Camellia sinensis*) in order to give insights on the popular trend in Taiwan, particularly during the summer months, of steeping tea leaves in cold water for approximately 2 h. Apparently, this method leads to lower amounts of caffeine, reduced bitterness and enhanced aroma. We found that the brewing conditions of tea indeed influences the polyphenol content and in vitro antioxidant capacity of the infusions. This was especially evident for white tea, which produced cold infusions with consistently higher polyphenol content and antioxidant capacity than the traditionally prepared hot beverage [14,15,16,17]. 

With this background knowledge, in the present study, we attempt to address the differences in the phenolic profile and antioxidant capacity between cold-brewed rooibos tea and brews prepared by either steeping in freshly boiled water or by boiling but are then chilled for consumption as a cold beverage. We simulated the preparation of a cup of rooibos to reflect consumer habits, using either cold water or hot water, including the practice of modern consumers that entails boiling of the teabag in water in a household microwave to improve extraction. Traditionally, the brew was kept on a hot stove for an extended period for better extraction, but use of a microwave is more convenient. The ORAC (oxygen radical absorbance capacity), ABTS (2,2’-azinobis-(3-ethylbenzothiazoline-6-sulfonic acid) diammonium salt) and Folin-Ciocalteu assays were used for determining the in vitro antioxidant capacities and total polyphenol content of the brews, as these are the most commonly used assays for analysis of foods and dietary supplements based on their advantages and disadvantages [18,19]. The relevance of these methods as first level screening of potential bioactivities of plant foods has recently been contextualized in terms of the reduction in the activation of NF-κB [20]. In the present study, a comprehensive evaluation of the various phenolic constituents was performed, using an untargeted metabolomics approach to putatively annotate the phenolic compounds released from rooibos leaves and stems during the three different brewing methods. Ultra-high-performance liquid chromatography-quadrupole time-of-flight (UHPLC-QTOF) mass spectrometry with electrospray ionization was used as analytical tool and Phenol-Explorer as database for putative annotation of compounds. In this study, both red and green rooibos were considered, since green rooibos, although less popular than the conventional red version, is of increasing interest. This interest derives from its higher antioxidant content [21]. Apart from the obvious difference in aspalathin content, green rooibos also shows a different phenolic profile when analyzed by 2-dimensional chromatography [22,23]. Overall, the results of this study will aid understanding of the impact of the preparation method on the potential health benefits of this unique beverage as indicated by its phenolic profile and in vitro antioxidant capacities.

## 2. Materials and Methods 

### 2.1. Chemicals and Equipment

All chemicals were of the highest analytical grade: 2,2’-azinobis-(3-ethylbenzothiazoline-6-sulfonic acid) diammonium salt (ABTS), 6-hydroxy-2,5,7,8-tetramethylchroman-2-carboxylic acid (Trolox), 3,4,5-trihydroxybenzoic acid (gallic acid), Folin-Ciocalteu reagent (2N solution), potassium persulfate (K_2_S_2_O_8_), sodium carbonate, 3′,6′-dihydrosyspiro[isobenzofuran-1[3H],9′[9H]-xanthen]-3-one (fluorescein), 2,2′-azobis(2-methylpropionamidine) dihydrochloride (AAPH) were purchased from Sigma-Aldrich Chemical Co. (Milan, Italy). Polyphenol standard compounds (> 98% purity) were purchased from Extrasynthèse (Genay, France). Ultrapure water was generated from a Milli-Q system by Merck Millipore (Merck KGaA, Darmstadt, Germany) and was used for all the experiments. Spectrophotometric measurements were recorded on a microplate reader (Synergy HT, Biotek, Winooski, VT, USA).

### 2.2. Rooibos Samples and Preparation of Aqueous Infusions

Twenty-four individual production batches of South African, unpasteurized green and red rooibos (*Aspalathus linearis*), 12 of each type to take into account batch-to-batch compositional variations, were used in this study. Green rooibos samples were collected from the same plantation and were kindly supplied by Rooibos Ltd. (Clanwilliam, Western Cape, South Africa). Four of these samples were loose leaf cut (less stems) compared to the rest that were fine cut (but with more white stems). Red rooibos samples were from two production areas, Western Cape (Rooibos Ltd., Clanwilliam,) and Northern Cape (Bokkeveld Rooibos, Nieuwoudtville, South Africa) and of the two top quality grades, A and B. Grade A presents the higher quality and grade B the bulk of red rooibos production. Grading is based on sensory characteristics (leaf color, infusion color and flavor) [7] and the percentage yield of refined rooibos (obtained after sieving). All samples were mechanically sieved and the fraction (particle size < 1.68 mm; > 0.42 mm) used for analysis.

**Figure 1:**
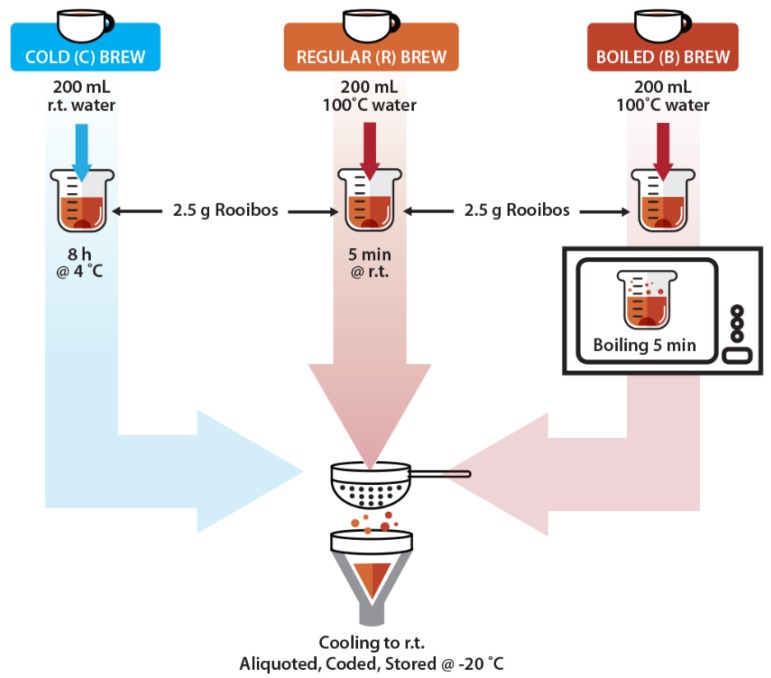
A graphical outline of the three different brewing methods used for the preparation of both red and green rooibos herbal tea, equalling a ‘one-cup-serving’.

Three different infusions were prepared from each sample as depicted in Figure 1 using in each case, 2.5 g of rooibos plant material (green or red) and 200 mL of water, which represent the average weight of plant material contained in a tea bag or in a heaped teaspoon and the average volume of a cup of tea, as also standardized by the rooibos industry for a ‘one-cup-serving’. The cold brews (C) were prepared by pouring room temperature water (± 21 °C) over the plant material in a glass jar and stirring for 5 s with a stainless-steel spoon. The jar was closed with a lid and the infusion was left to steep for 8 h in a refrigerator (0–5 °C). The brew was then stirred again for 5 s, strained through a fine mesh strainer and filtered through Whatman No. 4 filter paper. The regular brews (R) which simulate the most common method for preparing a hot cup of rooibos, were prepared by pouring boiling water (100 °C) over the plant material in a glass jar and stirring for 5 s. The container was closed, and samples were left to steep for 5 min at room temperature (± 21 °C). The infusion was stirred again for 5 s, strained through a fine mesh strainer and filtered through Whatman No. 4 filter paper. The boiled infusions (B) were prepared in a similar manner to the regular brews, but in a microwavable 1 L glass jug (to prevent the brew from boiling over in the microwave oven). After pouring boiling water (100 °C) over the plant material, followed by 5 s stirring, a lid was placed on the jug and the brew was boiled in a microwave oven on high for 5 min (2450 MHz; microwave input power 1400 W; microwave energy output 1000 W). The brew was stirred again for 5 s before straining and filtering, as described above.

All the above infusions were aliquoted and stored at −20 °C until analyzed. Each brewing method was performed in triplicate for each sample.

### 2.3. Determination of In Vitro Antioxidant Capacity (ABTS, ORAC)

The in vitro antioxidant capacity was evaluated by means of two different methods, namely the ABTS and ORAC (oxygen radical absorbance capacity) assays, as previously described in [24] and tested in triplicate (*n* = 9). The results from each assay are expressed as mmol Trolox equivalents per litre (mmol TE/L) using the linear regression obtained from a Trolox calibration curve.

The colored radical cation (ABTS•^+^) was prepared by mixing a 7.0 mM aqueous ABTS solution with a 24.5 mM aqueous solution of K_2_S_2_O_8_ as oxidizing agent in a 9:1 ratio respectively and allowing the mixture to stand at room temperature in the dark for 12–16 h before use. The prepared ABTS•^+^ stock solution was then diluted ≈50-fold with water to reach an absorbance of 0.9 ± 0.1 at 734 nm. For this assay, 30 μL of each brew previously diluted 60x with water, or of a 1.8 mM Trolox standard ethanolic solution appropriately diluted (0–0.30 mM in water), or water as control, were added in each well of a transparent 96-well microplate, followed by 270 μL of the diluted ABTS^•+^ solution. The microplate was shaken and left to stand for 120 min at room temperature in the dark; the absorbance of the solution was then read at 734 nm against water as blank. The antioxidant activity was determined as inhibition percentage.

For the ORAC assay, in each well of a solid black 96-well microplate, 25 µL of rooibos infusions previously diluted 450x with PB (phosphate buffer 75 mM, pH 7.4), or of an 18.0 mM Trolox standard ethanolic solution appropriately diluted (0.01–0.1 mM in PB), or PB as control, were mixed with 150 µL of a 0.008 μM solution of fluorescein in PB. After 30 min incubation in the dark at 37 °C, 75 μL of a 25 mM AAPH solution in PB were rapidly added to each well and fluorescence was recorded from above every 120 s for 3 h, using an excitation wavelength of 485/20 nm and an emission filter of 528/20 nm. The kinetics showed a classic fluorescence decay due to bleaching of fluorescein that was delayed in the presence of rooibos samples or of Trolox standard solution. AUC (area under the fluorescence decay curve) was automatically calculated by the analytical software Gen5 2.00.18 (Biotek, Winooski, VT, USA) connected to the Synergy HT microplate reader. The net AUC for each standard/compound was obtained by subtracting the area of the control sample (PB) from each one.

### 2.4. Determination of Total Phenolic Content (TPC)

The TPC of the rooibos brews was determined using the Folin-Ciocalteu reagent, as previously described in [24]. Briefly, 50 μL of each infusion previously diluted 15x with water, or of a 60 mM gallic acid standard ethanolic solution appropriately diluted (0–0.64 mM in water), were transferred in each well of a transparent 96-well microplate. Then, 150 μL of a 10-fold diluted solution of the Folin–Ciocalteu reagent was added. The microplate was shaken and left to stand for 10 min in the dark before adding 100 μL of a 10% Na_2_CO_3_ aqueous solution to each well. Samples were left to stand for 120 min at room temperature in the dark and then absorbance was read at 760 nm against water as blank. The brew of each sample was tested in triplicate (*n* = 9). The results are expressed as mg gallic acid equivalents per litre (mg GAE/L), using the linear regression deriving from the gallic acid calibration curve.

### 2.5. UHPLC-ESI-QTOF-MS Phenolic Profiling

The comprehensive phenolic profile of each sample was investigated by means of UHPLC-QTOF mass spectrometry, according to previously optimized analytical conditions [25,26]. Briefly, the mass spectrometer worked in positive scan mode to acquire masses in the range 50–1200 *m/z*. Chromatographic separation was achieved using an Agilent Zorbax Eclipse-plus C18 column (100 × 2.1 mm i.d., 1.8 μm) and a mixture of water and acetonitrile (LC-MS grade, VWR, Milan, Italy) as a mobile phase, both acidified with formic acid (0.1%, *v/v*). The injection volume was 4 μL, while the gradient was from 6% acetonitrile to 94% acetonitrile within 33 min, using a flow rate of 220 μL/min. Agilent Profinder B.06 software was used to process raw spectral data, according to the targeted ‘find-by-formula’ algorithm. The highest confidence in the designated annotation was achieved using monoisotopic mass information together with the isotopic pattern (both isotopic spacing and ratio) and adopting a 5-ppm tolerance for mass accuracy. To this end, the comprehensive database Phenol-Explorer 3.6 [27] was used as a reference for annotation. The metabolomic workflow used allowed compound identification according to Level 2 (i.e., putatively annotated compounds), with reference to COSMOS Metabolomics Standards Initiative [28,29]. Thereafter, mass and retention time alignment and filter-by-frequency (features not present in at least one treatment in 100% of replications were discarded) were applied. However, when multiple annotations were possible, we selected those phenolic compounds previously identified in rooibos [30,31,32,33,34,35]. To achieve a higher degree of confidence in annotation, pooled samples were further analyzed in tandem mass spectrometry, using the previously reported conditions and a data-dependent MS/MS approach in place of the MS-only SCAN. With this aim, a precursor absolute threshold of 5000 and relative threshold of 0.01%, with a maximum of 12 precursors per cycle and active exclusion set at 2 spectra, were adopted. Compound annotation was then performed using the software MS-DIAL 3.98 [36], using all publicly available MS/MS records built in the same software.

Finally, the phenolic compounds ascribed to classes and sub-classes were quantified using standard solutions (80/20, *v/v* methanol/water) of pure authentic phenolic compounds analyzed with the same method [25]. The following phenolic standards were used: cyanidin (for anthocyanins), luteolin (for flavones, flavanones, isoflavonoids, chalcones, and dihydrochalcones), catechin (for flavanols and flavonols), matairesinol (for lignans), ferulic acid (for phenolic acids), resveratrol (for stilbenes) and tyrosol (for the other remaining phenolics). Calibration curves were built using a linear fitting (un-weighted and not forced to axis-origin) in the range 0.05–500 mg/L; a coefficient of determination *R^2^* > 0.99 was used as acceptability threshold for calibration purposes. The results were expressed as mg/L phenolic equivalents.

### 2.6. Statistics and Chemometrics 

One-way analysis of variance (ANOVA) was carried out with JMP software for total phenolic content (TPC) and in vitro antioxidant capacity values (ABTS and ORAC). The Tukey’s multiple comparison test (*p* < 0.01) post-hoc analysis was used to compare means. Pearson’s correlations (p = 0.01, two-tailed) were also calculated using IBM SPSS software (Version 25.0). 

Metabolomics-based data were analyzed using the Agilent Mass Profiler Professional B.12.06 software. Data normalization was achieved as previously reported [25,26]. Afterwards, SPSS software was used to elaborate semi-quantitative values for each phenolic class/sub-class, with a one-way ANOVA (*p* < 0.05; Duncan’s post-hoc test). Subsequently, the dataset was exported into SIMCA 13 (Umetrics, Malmo, Sweden) for Orthogonal Projections to Latent Structures Discriminant Analysis (OPLS-DA) modelling, investigating also the presence of outliers and a possible overfitting (i.e., Hotelling’s T2 and permutation testing). OPLS-DA model parameters (R^2^Y and Q^2^Y) were also inspected. Finally, the variable selection method VIP was used to depict the most discriminant phenolic compounds, selecting those with the highest score (VIP score > 1). 

## 3. Results 

### 3.1. Effect of Hot and Cold Brewing on Antioxidant Capacity and Total Polyphenol Content of Red and Green Rooibos

**Figure 2:**
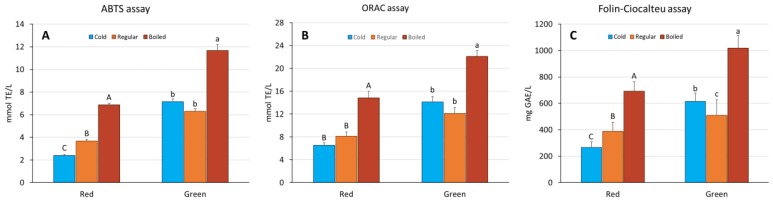
Effect of brewing method on the antioxidant capacity measured using the ABTS assay (**A**) and ORAC assay (**B**), and on Total Polyphenol Content (**C**) according to the Folin-Ciocalteu assay of red (fermented), and green (unfermented) rooibos tea. Colored bars indicate the type of brew: Blue = Cold, Orange = Regular, Red = Boiled. Error bars represent ± SD, *n* = 12. Tukey’s post-hoc multiple comparison test (*p* < 0.01) was made separately for red and green rooibos tea (in letters).

The results obtained for both red and green rooibos brews using the ABTS assay are depicted in Figure 2A. Boiling (B) clearly resulted in brews having a significantly higher antioxidant capacity (green = 11.7 mmol TE/L and red = 6.9 mmol TE/L) than cold (C) and regular (R) brewing, but the difference between the cold and regular brews was not significant (*p* > 0.05) for green rooibos (i.e., 7.1 mmol TE/L vs 6.3 mmol TE/L, respectively). However, cold-brewed red rooibos had a significantly lower (*p* < 0.01) antioxidant capacity than its regular brew (R) (i.e., 2.4 mmol TE/L vs 3.7 mmol/L TE, respectively). Concerning inter-batch variations (see Supplementary Materials) it can be noted that for the green rooibos samples, the brewing method plays a relevant role; the four long cut samples indeed showed approximately half the levels of antioxidant capacity than the fine cut samples when prepared with the common, regular hot brewing method (R), but these inter-batch differences were less noticeable in the boiled brews (B) and were not observed at all in the cold brews (C). 

A similar trend outlined above was also observed for the data generated using the ORAC assay (Figure 2B) for both red and green rooibos: indeed, a strong significant correlation was found between these two assays (0.977; *p* < 0.01). The boiled brews (B) had a significantly higher antioxidant capacity (green = 22.1 and red = 14.9 mmol TE/L) than the cold (C) and regular (R) brews. The latter brews did not differ significantly (*p* > 0.05) from each other (green: R = 12.1 and C = 14.1; red: R = 8.1 and C = 6.6 mmol TE/L). The results obtained for TPC (Figure 2C) follow a similar pattern to those observed with the two antioxidant assays. In fact, the TPC results strongly correlate with those of both ORAC and ABTS assays (ABTS: 0.953, ORAC: 0.951; *p* < 0.01). In detail, the green rooibos brews contain 1019.0 (B), 508.7 (R) and 614.1 (C) mg GAE/L, while the corresponding content values for red rooibos were 692.4, 387.9 and 265.4 mg GAE/L. However, to gain further insight into the impact of the different brewing methods on both the polyphenol content and antioxidant capacity, fingerprinting of the phenolic profile was carried out by using an untargeted metabolomics approach.

### 3.2. Untargeted Phenolic Profile and Discrimination of Different Samples

The phenolic composition of the rooibos brews was evaluated using a high-resolution untargeted UHPLC-QTOF mass spectrometric approach. This allowed putative annotation of 187 compounds including 87 flavonoids, 43 tyrosol equivalents, 35 phenolic acids, 16 lignans, and 6 stilbenes (Supplementary Materials). Some of the major and other minor flavonoids of rooibos, including the dihydrochalcone, aspalathin, the flavanones, naringenin 6-*C*-glucoside and hemiphlorin, the flavones, luteolin 7-*O*-glucoside (luteoloside), luteolin-8-*C*-glucoside (orientin) and luteolin-6-*C*-glucoside (isoorientin) and the flavonols, quercetin and quercetin glycosides, were also putatively annotated. The samples were found to be rich in phenolic acids, such as caffeic, ferulic, *p*-coumaric and *p*-hydroxybenzoic acids, previously identified by Rabe et al. [34]. Besides, a dedicated tandem-MS approach allowed us to confirm, among the others, the identity of vitexin (apigenin 8-*C*-glucoside), orientin (luteolin 8-*C*-glucoside), quercetin 4-*O*-glucoside, rutin and chrysoeriol, as seen in the Supplementary Materials. 

Thereafter, by using a semi-quantitative analysis from the UHPLC-QTOF data, the ‘identified’ phenolic compounds were classified and reported according to a representative standard per class/sub-class as mg/L equivalents. The results of the semi-quantitative analysis are reported in Table 1 for both green and red rooibos brews. 

According to this analysis, we found that the boiled (B) green rooibos brews were characterized by the highest (*p* < 0.05) polyphenol content (714.4 mg/L), followed by the cold (C)- (628.5 mg/L) and regular (R)- brewed samples (597.6 mg/L). Overall, the boiled brews were characterized by higher apparent content values for ferulic acid, matairesinol, catechin and tyrosol equivalents compared to the other samples, recording values of 108.1, 17.8, 83.9 and 250.7 mg/L, respectively. No significant differences were observed among the different brewing conditions when considering the apparent content values for anthocyanins (on average: 43.1 mg/L) and stilbenes (on average: 8.4 mg/L). Similar information was obtained by inspecting the semi-quantitative results for red rooibos; however, in this case, boiling was found to strongly enhance the extraction of luteolin (on average: 110.9 mg/L) and anthocyanin (on average: 21.0 mg/L) equivalents. Overall, the semi-quantitative analysis following untargeted profiling of rooibos tea revealed that the boiled (B) brewing method resulted in the highest total phenolic content, with four phenolic class equivalents, namely luteolin, catechin, tyrosol and ferulic acid, mainly affected (Table 1). Therefore, these findings clearly show the impact of temperature on the final polyphenol content of the brew. Besides, in accordance with the literature [23,37], the aspalathin content (evaluated as luteolin equivalents) was 10-fold higher in green rooibos tea than in red tea, being 79.5 vs 6.6 mg/L equivalents, respectively (Supplementary Materials).

**Figure 3:**
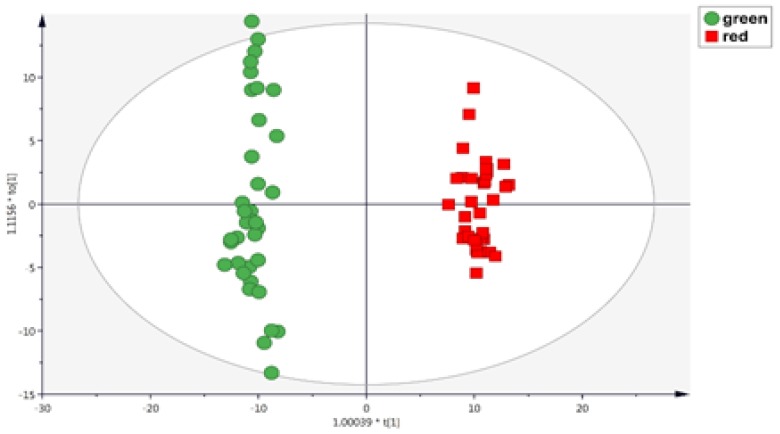
OPLS-DA score scatter plot obtained considering green (*n* = 36) and red (*n* = 36) rooibos tea phenolic profiles, independent from the brewing method used.

Subsequently, considering that the main differences were represented in the metabolomic dataset, a supervised OPLS-DA multivariate statistical approach was used to identify the contribution of each (poly)-phenolic group for discrimination purposes, according to the different processing and preparation conditions. The OPLS-DA score plot (Figure 3) shows clear differences between red and green rooibos samples, independently from the brewing method used. The model parameters are characterized by high values for goodness-of-fit (R^2^Y = 0.98) and goodness-of-prediction (Q^2^Y = 0.98).

Thereafter, in order to evaluate the ‘variable importance in projection’ of the OPLS-DA model, the VIP approach was exploited. The putatively annotated compounds are reported in the Supplementary Materials with their prediction score (VIP score > 1). This approach allowed identification of 73 compounds able to discriminate green rooibos from red rooibos, including 41 flavonoids (i.e., 11 anthocyanins, 11 flavones and 10 flavonols), 12 phenolic acids (mainly hydroxycinnamic acids), 5 lignans and 2 stilbenes. Interestingly, the VIP approach highlighted aspalathin as the most discriminant compound in the model, possessing a VIP score of 1.74, thus confirming the impact of the ‘fermentation’ of the plant material on its final content.

**Figure 4:**
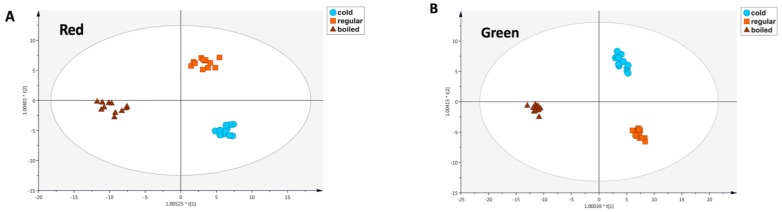
OPLS-DA score scatter plots obtained considering the phenolic profiles of red (**A**) and green (**B**) rooibos tea, as resulting by cold (*n* = 12), regular (*n* = 12) and boiled (*n* = 12) brewing methods.

A second combined OPLS-DA model was then built to highlight the differences between the cold and hot brews, considering the red and green rooibos samples separately. For red rooibos, the prediction model clearly discriminated the different groups, i.e., ‘cold’, ‘boiled’ and ‘regular’ (Figure 4A), with the model values being more than acceptable, recording R^2^Y = 0.97 and Q^2^Y = 0.83. In particular, the second latent vector suggested that boiled-brewed samples were completely different from the cold- and regular-brewed samples, thus corroborating the semi-quantitative results previously reported. 

Thereafter, the VIP selection method was used to identify the compounds able to discriminate between ‘cold’ vs ‘boiled’ and ‘cold’ vs ‘regular’ brewing, in order to identify the most affected phenolic compounds. Considering the comparison ‘cold’ vs ‘boiled’ brews, 32 discriminant polyphenols were considered as markers, mainly characterized by flavonoids and low-molecular-weight phenolics, that are reported in Table 2 together with their VIP score (> 1) and LogFC values (resulting from Fold-Change analysis; FC > 2). The same approach was used to explore the phenolic compounds most affected when considering the comparison ‘cold’ vs ‘regular’; the results of this second investigation are reported in Table 3. 

As can be observed, 35 phenolic markers were able to explain the differences observed, including a great abundance of flavonoids (mainly flavonols and flavanols). Overall, the LogFC values reported in Table 2 and Table 3 for red rooibos samples can be considered indicative of the extraction of these compounds as promoted by the different preparation methods used (i.e., regular, cold and boiled). Interestingly, the phenolic markers showing the highest up-accumulation values for the comparison ‘cold’ vs ‘boiled’ were myricetin-3-*O*-arabinoside (LogFC = 3.83) and piceatannol (LogFC = 2.80), whilst those characterizing the comparison ‘cold’ vs ‘regular’ were 3-hydroxybenzoic acid (LogFC = 10.91) and cyanidin-3-*O*-(6’’-acetyl-galactoside) (LogFC = 6.38). Subsequently, the same statistical approach was used to inspect the best phenolic markers for green rooibos samples (Figure 4B). This second OPLS-DA model was also characterized by good indicators for goodness-of fit (R^2^Y = 0.98) and goodness-of-prediction (Q^2^Y = 0.88). Interestingly, the comparisons previously reported provided a different number of discriminant compounds; in fact, a higher number of VIP markers was detected for the comparison ‘cold’ vs ‘boiled’ (44 markers; Table 2) compared to ‘cold’ vs ‘regular’ (27 markers; Table 3). Overall, the most abundant class was that of flavonoids, followed by phenolic acids. As can be observed from Table 2, the most important polyphenols discriminating ‘cold’ vs ‘boiled’ conditions were peonidin-3-*O*-galactoside (LogFC = 5.32) followed by *p*-coumaroyl glycolic acid (LogFC = 3.59), whilst those discriminating ‘cold’ vs ‘regular’ brewing methods were apigenin-6,8-*C*-galactoside-*C*-arabinoside (LogFC = 2.51) and sinapic acid (LogFC = 2.02). It is also important to underline that multivariate statistics revealed few differences between cold- and regular-brewed rooibos samples, with a low number of discriminant compounds strictly related to the cold brew (Table 3), independently from the oxidation status of the plant material (i.e., red vs green). 

Finally, the relationship between the quantitative profiles of the different polyphenol classes/sub-classes and the in vitro antioxidant capacities of green and red rooibos brews were statistically analyzed. With this aim, the Pearson’s correlation coefficients (Supplementary Materials) were inspected to determine possible linear relationships between the parameters under investigation. Overall, a significant correlation among all the studied parameters was observed (*p* < 0.01). In this regard, each phenolic class was found to contribute to the antioxidant capacity values (i.e., ABTS and ORAC). However, phenolic acids were those compounds recording the highest correlation coefficients with both ABTS and ORAC values, 0.88 and 0.84, respectively (*p* < 0.01). In addition, the total phenolic content from UHPLC-QTOF-MS analysis was found to strongly correlate with ORAC values (0.88; *p* < 0.01) and TPC values as determined by the Folin-Ciocalteu assay (0.81; *p* < 0.01). Interestingly, strong correlations were also observed between all the flavonoid sub-classes considered (i.e., anthocyanins, flavonols, flavanols and flavones). Previously, Orzel et al. [38] showed that total antioxidant capacity values obtained from the ORAC and DPPH assays of regular-brewed rooibos tea could be predicted from the chromatographic fingerprints (prediction errors 9–12%) using partial least squares (PLS) regression, corroborating our findings using UHPLC-QTOF-MS fingerprinting of phenolic compounds.

## 4. Discussion

The aim of the present study was to investigate if, and to what extent, cold-brewed rooibos differs from the regular hot and boiled brews in terms of antioxidant capacity and (poly)phenolic profile, an aspect that has so far been overlooked by the scientific community. This was mainly carried out in view of the surging popularity of rooibos tea-drinking outside its native country, especially in Japan, where it is common to prepare and serve it also as a cold brew, i.e., the use of hot water or boiling is eliminated. The cold brew is particularly popular in the hot and humid summer months due to its ease of preparation and fresher, sweeter, lighter-bodied flavor and taste profile (anecdotal evidence) since the plant material is extracted much slower. In this study, we also included a boiled brew as prepared by the modern consumer, using a household microwave. Traditionally, a strong cup of rooibos tea was prepared by slowly simmering the leaves and stems in a pot on a hot stove [30], but this practice later fell in disfavour with the introduction of tea bags in the 1960s and convenience becoming paramount when following a rushed lifestyle.

In this work, the differences in terms of in vitro antioxidant capacity between the different brewing methods applied to green and fermented rooibos were determined using two commonly used methods, namely ABTS and ORAC assays. These methods differ in their determination principles, but they are able to provide a comprehensive picture of the antioxidant potential of the samples under investigation when combined opportunely [39]. The results reported here indeed show that cold-brewed rooibos differs in antioxidant capacity and phenolic profile compared to the hot brews, and that the different brewing conditions have a different impact depending on the oxidation status of rooibos plant material considered. Concerning the more popular red rooibos; cold brewing (C) compared to the common regular brewing (R) method reduced the antioxidant capacity of the brew. However, for maximum benefits within the context of the preparation methods investigated, boiling (B) in a microwave for a short time (5 min) would appear to be the best choice since it produced brews having twice the TPC and in vitro antioxidant capacity levels than when regular brewing was employed. A previous study showed that additional heat treatment of red rooibos extracts was not detrimental to the ability of the extract to protect against lipid peroxidation [40]. However, for green rooibos, the cold brew had surprisingly higher TPC and antioxidant capacity values, although not significantly higher, compared to the regular brew. This finding is of particular interest as it indicates that cold brewing of green rooibos does not affect the in vitro antioxidant properties of the beverage compared to the regular hot brew, an interesting aspect as it is expected to impact on aroma, flavor and taste. This aspect is still to be investigated. In addition, leaving the plant material to steep for 5 min in freshly boiled water, as for the regular hot brewing method (R), may not be long enough for uniform extraction of polyphenols among the different batches of plant material, since differences were observed. Instead, the inter-batch variations did not manifest to such an extent in cold-brewed green rooibos as observed for the hot regular brew (R). Most likely, the 8 h duration of cold brewing appears to be sufficiently long to fully extract the bioactive polyphenols relatively equally from the different batches of leaves despite the size of the particulate matter. The differences among the batches can still be observed even when the plant material is boiled for 5 min (B), although to a lesser extent. The inter-batch variations are mainly related to the percentage of ‘leaf needle’ or leaf-to-stem content of the plant material mixture, as well as to the size of the cut, which would affect mass transfer through diffusion, and can thus be influenced by the method of extraction as previously reported for other teas [15].

Our results clearly indicate that the brewing temperature for the two hot preparation methods, regular (R) and boiled (B) brewing, is a major factor leading to the above outcomes, since the extraction time is the same (5 min), but the temperature is different (non-constant vs constant 100 °C, respectively) [41,42]. Furthermore, microwave dielectric heating compared to conventional heating accelerates energy transfer and facilitates penetration of the solvent into the matrix and solvation of plant constituents, resulting in more efficient extraction [43]. Clearly, the brewing temperature is also a factor when comparing the cold brew with the boiled brew for both red and green rooibos. However, for green rooibos this factor does not appear to be as dominant, since the cold and regular brews had similar TPC and antioxidant capacity values, indicating that brewing time is a deciding factor. For red rooibos, both time and temperature are important, since significant differences are evident between the two brewing methods. Santos et al. [11] observed that red rooibos extracted at the highest temperature examined had the highest antioxidant capacity and phenolic content and that extraction time was less important. However, if the temperature is kept constant, steeping time does become relevant, as demonstrated recently [10]. A similar conclusion was also reached by McAlpine et al. [8] who studied the effects of steeping time at constant 96 °C on the TPC and antioxidant capacity of green and red rooibos in loose ‘leaf’ format. They demonstrated that the TPC values increased with longer durations of steep time with > 50% polyphenols extracted after 5 min. A follow-up study by the same authors using rooibos in tea bags instead of loose format, showed that the majority of polyphenols was also extracted after 5 min [12]. Marnewick et al. [44] showed that daily consumption of 6 cups of red rooibos for a duration of 6 weeks, with a cup of rooibos brewed under the same conditions as our regular brew (R), significantly improved the blood lipid profile (serum low-density/high-density LDL/HDL-lipoprotein cholesterol, triacylglycerols), as well as redox status (reduced glutathione:oxidized glutathione GSH:GSSG ratio), of adults at risk for cardiovascular disease. No dose optimization was attempted for the latter study. Considering the results reported here, the higher bioactive phenolic content and antioxidant capacity obtained by boiling rooibos leaves for at least 5 min in a microwave, could potentially reduce the number of cups of rooibos a day required for an effect. This is still speculative and a very simplistic assessment as it does not take into account the inherent variation in the levels of specific bioactive compounds, such as aspalathin, and needs further consideration. The choice of a marker parameter to assess the apparent equivalence of a cup of rooibos was previously highlighted for red rooibos extracts and could lead vastly different equivalences [45]. Furthermore, in summer, more than 6 cups of cold-brewed tea can be easily consumed instead of water, thanks also to its ease of preparation, in order to improve health status.

Although the aim of our study was not to compare the antioxidant capacity nor phenolic profile of green and red rooibos as this is a well explored area, but mainly to compare cold- vs hot-brewed rooibos for both types of tea, our results confirm that the green rooibos brews have approximately a two-fold higher antioxidant capacity compared to red rooibos brews, because of its higher polyphenol content [10,37,46,47]. Furthermore, the strong correlation values found between the antioxidant assays and TPC indicate that the main contributors to the antioxidant activity of rooibos tea, whether red or green, are the polyphenols and are in line with the data reported in the literature [21]. However, from our results, it appears that boiled red rooibos tea has similar antioxidant capacity to cold- and regular-brewed green rooibos. Once again, this could be an oversimplification of the potential health benefit accrued by manipulating brewing as it does not take into account compositional differences with the accompanied differences in bioavailability. For example, aspalathin and quercetin are equally potent antioxidants in vitro [48], yet quercetin should be more bioavailable considering the number of violations of Lipinski’s ‘Rule of 5’, and other parameters such as the number of rotatable bonds and polar surface area (PSA) [49,50,51]. Aspalathin indeed has low bioavailability [52].

Several analytical methods suitable for the simultaneous quantification of selected rooibos phenolics have been reported in the literature [5,23,53]. Although an untargeted metabolomic approach has recently been reported on red rooibos extracts [54], this is the first time that such an approach has been carried out on both green and red rooibos infusions, prepared using three typical ‘household’ methods for preparation of a ‘one-cup-serving’. The most recent report in this field [55] on methanol/water extracts of fermented rooibos from ‘wild-tree’ ecotypes using a targeted method by UHPLC-ESI-QTOF-MS, stated that 59 compounds were detected (of which 50 were identified). Walters et al. [56] could (tentatively) identify 39 phenolic compounds, of which, 18 have no previously been reported in rooibos, using two-dimensional chromatography and UV-Vis, MS and MS/MS data. However, in the other available studies, authors mainly used targeted analytical approaches. For example, some authors detected and identified up to 28 phenolic compounds in aqueous infusions of green and red rooibos by LC-ESI-MS and LC-ESI-MS/MS [53]. In another report, Iswaldi et al. [33] identified 25 phenolic compounds using HPLC-ESI-MS in aqueous extracts of red rooibos prepared by boiling 20 g of rooibos leaves in 100 mL of distilled water for 5 min. In addition, they reported that orientin and isoorientin were the predominant compounds. In the present study, despite the untargeted nature, the UHPLC-ESI-QTOF-MS approach used was capable of detecting 187 compounds belonging to different classes/sub-classes as reported in the Supplementary Materials, hence over 100 new compounds could be putatively annotated, based on the available data in Phenol-Explorer. Further investigation is needed to confirm the identity of the putatively annotated compounds not previously found in rooibos. However, we also confirmed, by means of a tandem-MS approach, the identity of orientin (luteolin-8-*C*-glucoside), vitexin (apigenin-8-*C*-glucoside), quercetin-4-*O*-glucoside and chrysoeriol (Supplementary Materials), which are some of those compounds characterizing both green and red rooibos according to the literature [55] (Supplementary Materials). Therefore, despite the need to confirm compound identification in more detail, the untargeted metabolomic approach was able to highlight that brewing conditions affect not only the quantity of polyphenols extracted, which were always higher in boiled brews, followed by the regular and cold brews, for both rooibos types, but also qualitative differences, which could impact on the ‘quality’ of the bioactive phenolic profile. Indeed, the OPLS-DA models followed by the VIP selection method, for both red and green rooibos, revealed most likely that some heat-sensitive components in cold brews are not degraded or remain unaltered, compared to what happens during boiled brewing. For example, the VIP selection method following OPLS-DA analysis outlined 39 up-accumulated discriminant compounds when considering the comparison cold vs boiled brews (combining green and red rooibos; Table 2). Among these compounds, an abundance of flavonoids (mainly flavonols), followed by low-molecular-weight compounds (i.e., phenolic acids and tyrosol equivalents) was noted. These findings are interesting, since one could tailor a rooibos beverage simply by changing the brewing method and type of rooibos tea used, whether green or red. Overall, the untargeted findings outlined by metabolomics show that the oxidation status (fermented and unfermented) of the product is the main discriminant factor able to affect the polyphenol content, as also observed in previous studies [46,53,56,57,58]. However, it is possible to postulate that the type of brewing method can also modulate the bioactive composition and the corresponding health-promoting properties of rooibos tea.

## 5. Conclusions

The health-promoting claims for rooibos, in addition to its pleasant aroma, flavor and taste, and caffeine-free status, contribute to its increasing wide appeal. However, rooibos is competing in a market encumbered with products endowed to be ‘rich in antioxidants’. Hence it is important in the face of such competition to determine how to maximize the benefits of a cup of rooibos. The results presented here indicate that cold-brewing green rooibos tea delivers the same TPC and antioxidant capacity as the regular hot brew, unlike for red rooibos, with cold brewing resulting in slightly lower levels of both parameters. However, in the context of this study maximum potential health-promoting benefits could be achieved, regardless of rooibos type, by boiling in a microwave. Overall, our findings suggest that if rooibos tea is consumed for its particular health outcomes rather than its sensory profile, then the differences in antioxidant capacity and polyphenol profile encountered among the three ‘household’ preparation methods, should be considered. For the first time, an untargeted metabolomic approach of both green and red rooibos has detected 187 phenolic compounds in their brews, and a comprehensive phenolic profile of both red and green rooibos is presented, highlighting the differences between the cold and hot brews. Future research should address confirmation of the identity of the putatively annotated compounds not previously found in rooibos.

## Figures and Tables

**Table 1:** Quantification per classes/sub-classes of phenolic compounds identified from UHPLC-QTOF-MS data for the different rooibos tea samples.

Matrix	Cyanidin Eq.	Luteolin Eq.	Catechin Eq.	Matairesinol Eq.	Tyrosol Eq.	Ferulic Acid Eq.	Resveratrol Eq.	Total
***Red Rooibos***								
**Cold brew**	12.4 ± 3.3^a^	75.4 ± 7.1^a^	36.2 ± 8.9^a^	11.8 ± 3.5	157.8 ± 10.7^a^	65.6 ± 7.2^a^	1.7 ± 0.6^a^	360.9 ± 25.5^a^
**Boiled brew**	21.0 ± 5.3^b^	110.9 ± 14.4^c^	43.9 ± 11.0^b^	12.0 ± 2.0	189.5 ± 20.9^b^	91.9 ± 5.4^b^	2.4 ± 0.7^b^	471.6 ± 33.5^c^
**Regular brew**	15.5 ± 3.1^a^	94.0 ± 14.9^b^	37.7 ± 11.2^a^	12.1 ± 2.4	161.1 ± 12.8^a^	63.8 ± 6.0^a^	1.7 ± 0.2^a^	385.9 ± 26.9^b^
*Significance*	***	***	***	ns	***	***	*	***
***Green Rooibos***								
**Cold brew**	42.3 ± 7.4	182.9 ± 10.9^a^	79.7 ± 14.8^ab^	13.4 ± 3.8^a^	217.5 ± 40.0^ab^	84.5 ± 8.0^b^	8.2 ± 1.2	628.5 ± 39.0^b^
**Boiled brew**	44.5 ± 9.2	200.4 ± 20.0^b^	83.9 ± 16.8^b^	17.8 ± 2.6^b^	250.7 ± 19.7^b^	108.1 ± 13.9^c^	9.0 ± 0.9	714.4 ± 42.0^c^
**Regular brew**	42.6 ± 9.9	187.1 ± 15.1^a^	77.1 ± 15.2^a^	12.3 ± 2.1^a^	203.9 ± 20.7^a^	66.5 ± 11.7^a^	8.1 ± 1.0	597.6 ± 48.6^a^
*Significance*	ns	*	*	**	*	***	ns	***

Results are expressed as mean values (mg phenolic equivalents/L) ± standard deviation (*n* = 12). Superscript letters within each column (considering green and red rooibos, separately) indicate homogeneous sub-classes as resulted from ANOVA (*p* < 0.05; Duncan’s post-hoc test). ns = not significant; *** = *p* < 0.001; ** = *p* < 0.01; * = *p* < 0.05. Eq. = Equivalents.

**Table 2:** Discriminant phenolic compounds according to the comparison ‘cold’ vs ‘boiled’ brewing and considering both green and red rooibos tea. Compounds were identified by the VIP (variable importance in projection) approach following OPLS-DA discriminant analysis and are provided together with VIP scores (measure of variable’s importance in the OPLS-DA model), LogFC values (obtained by Fold Change analysis) and accumulation.

	VIP Marker (OPLS-DA)	VIP Score	LogFC	Accumulation
***Green Rooibos***				
Flavonoids	Isorhamnetin-3-*O*-rutinoside; Chrysoeriol-7-*O*-glucoside	1.79 ± 0.36	−2.73	Down
	Formononetin	1.73 ± 0.46	−0.17	Down
	Cyanidin-3-*O*-galactoside	1.69 ± 0.55	−1.24	Down
	Fisetin; Luteolin	1.54 ± 0.64	−0.51	Down
	Kaempferol	1.54 ± 0.64	−0.51	Down
	Hispidulin; Chrysoeriol	1.50 ± 0.52	−2.39	Down
	8-Prenylnaringenin	1.41 ± 0.75	−1.91	Down
	Gardenin B	1.39 ± 0.54	−2.51	Down
	Hemiphlorin; Naringenin-6-*C-*glucoside; Naringenin-7-*O-*glucoside	1.23 ± 0.91	−0.03	Down
	Cyanidin-3-*O*-(6’’-caffeoyl-glucoside)	1.87 ± 0.24	−0.02	Down
	Petunidin-3-*O*-galactoside	1.18 ± 0.95	−0.81	Down
	Genistein	1.03 ± 0.73	−3.56	Down
	Peonidin-3-*O*-galactoside	1.61 ± 0.53	5.32	Up
	Daidzin	1.58 ± 0.72	0.07	Up
	Isorhamnetin-3-*O*-glucoside-7-*O*-rhamnoside	1.53 ± 0.64	0.44	Up
	Myricetin-3-*O*-arabinoside	1.47 ± 0.63	3.83	Up
	Kaempferol-3-*O*-acetyl-glucoside	1.46 ± 0.57	0.76	Up
	Petunidin-3-*O*-(6’’-acetyl-galactoside)	1.45 ± 0.77	0.45	Up
	Cyanidin-3,5-*O-*diglucoside	1.37 ± 0.53	0.05	Up
	Sakuranetin	1.31 ± 0.75	0.35	Up
	Chrysin	1.24 ± 0.84	1.40	Up
	Cyanidin-3-*O-*(6’’-acetyl-galactoside)	1.23 ± 1.03	0.39	Up
	3-Hydroxyphloretin 2’-*O*-xylosyl-glucoside	1.16 ± 1.19	1.03	Up
	6-Geranylnaringenin	1.11 ± 0.66	0.68	Up
	Quercetin 3,4’-*O*-diglucoside	1.09 ± 0.82	0.42	Up
	5,6-Dihydroxy-7,8,3’,4’-tetramethoxyflavone	1.08 ± 1.04	0.40	Up
	(-)-Epigallocatechin-3-*O*-gallate	1.01 ± 0.88	1.30	Up
	3-Methoxysinensetin	1.00 ± 0.85	0.75	Up
Lignans	Todolactol A	1.09 ± 0.72	−1.18	Down
	Dimethylmatairesinol	1.00 ± 1.17	0.80	Up
Other polyphenols	Sinapaldehyde; Caffeic acid ethyl ester	1.68 ± 0.52	−0.80	Down
	Rosmanol	1.32 ± 0.83	−0.89	Down
	Demethyloleuropein	1.15 ± 0.75	−1.26	Down
	Benzoic acid; 4-Hydroxybenzaldehyde	1.74 ± 0.65	2.38	Up
	Isopimpinellin	1.25 ± 0.44	0.26	Up
	*p*-Anisaldehyde	1.08 ± 0.51	0.08	Up
Phenolic acids	Caffeic acid ethyl ester; Sinapaldehyde	1.68 ± 0.52	−0.80	Down
	Protocatechuic acid-4-*O*-glucoside	1.33 ± 0.44	−3.41	Down
	3,4,5-Trihydroxycinnamic acid; Hydroxycaffeic acid	1.08 ± 0.74	−1.26	Down
	Benzoic acid; 4-Hydroxybenzaldehyde	1.74 ± 0.65	2.38	Up
	*p*-Coumaroyl glycolic acid	1.15 ± 1.22	3.59	Up
Stilbenes	Piceatannol	1.42 ± 0.79	2.80	Down
***Red Rooibos***				
Flavonoids	Chrysoeriol; Hispidulin	1.99 ± 0.65	−2.39	Down
	Quercetin-3-*O*-xyloside	1.53 ± 0.77	−0.84	Down
	Gardenin B	1.47 ± 0.65	−2.51	Down
	Fisetin; Luteolin	1.38 ± 0.60	−0.51	Down
	Kaempferol-3-*O*-(6’’-acetyl-galactoside)-7-*O*-rhamnoside	1.38 ± 0.69	−0.77	Down
	Delphinidin-3-*O-*galactoside	1.36 ± 0.93	−8.53	Down
	Kaempferol-7-*O*-glucoside	1.35 ± 0.87	−1.21	Down
	Peonidin 3-*O*-(6’’-*p*-coumaroyl-glucoside)	1.33 ± 0.78	−2.88	Down
	Isorhamnetin-3-*O*-glucuronide	1.32 ± 0.88	−0.19	Down
	Xanthohumol; Isoxanthohumol	1.32 ± 0.83	−7.87	Down
	Peonidin	1.32 ± 0.60	−1.60	Down
	Petunidin-3-*O*-rhamnoside	1.31 ± 0.97	−1.43	Down
	Cyanidin-3-*O*-galactoside	1.06 ± 0.82	−1.24	Down
	Cyanidin-3-*O*-(6’’-caffeoyl-glucoside)	1.00 ± 1.13	−0.02	Down
	Myricetin-3-*O*-arabinoside	2.16 ± 0.41	3.83	Up
	Daidzin	1.13 ± 0.94	0.07	Up
	Quercetin-3,4’*-O*-diglucoside	1.04 ± 1.43	0.42	Up
Other polyphenols	Rosmanol	2.02 ± 0.43	−0.89	Down
	Demethyloleuropein	1.74 ± 0.68	−1.26	Down
	4-Hydroxycoumarin	1.53 ± 1.11	−0.16	Down
	Esculin	1.22 ± 0.88	−0.25	Down
	5-Pentadecylresorcinol	1.36 ± 1.24	0.77	Up
	*p*-Anisaldehyde	1.35 ± 1.17	0.08	Up
	Oleoside dimethylester	1.32 ± 0.71	1.07	Up
Phenolic acids	Hydroxytyrosol	1.15 ± 0.96	0.26	Up
	Isopimpinellin	1.00 ± 1.62	0.26	Up
	3,4,5-Trihydroxycinnamic acid; Hydroxycaffeic acid	1.68 ± 0.66	−1.26	Down
	Protocatechuic acid-4-*O*-glucoside	1.04 ± 0.68	−3.41	Down
	4-Hydroxybenzoic acid	1.14 ± 1.0	0.19	Up
Stilbenes	Pallidol	1.49 ± 1.23	−1,07	Down
	Piceatannol	1.23 ± 1.23	2.80	Up

**Table 3:** Discriminant phenolic compounds according to the comparison ‘cold’ vs ‘regular’ brewing and considering both green and red rooibos tea. Compounds were identified by the VIP (variable importance in projection) approach following OPLS-DA discriminant analysis and are provided together with VIP scores (measure of variable’s importance in the OPLS-DA model), LogFC values (obtained by Fold Change analysis) and accumulation.

	VIP Marker (OPLS-DA)	VIP Score	LogFC	Accumulation
***Green Rooibos***				
Flavonoids	Formononetin	1.90 ± 0.53	−2.26	Down
	Isorhamnetin-3-*O*-glucuronide	1.46 ± 0.78	−1.06	Down
	Galangin; Genistein	1.56 ± 0.78	−0.83	Down
	Xanthohumol; Isoxanthohumol	2.13 ± 0.47	−0.80	Down
	Hispidulin; Chrysoeriol	1.08 ± 0.87	−0.76	Down
	Isorhamnetin-3-*O*-rutinoside; Chrysoeriol-7-*O*-glucoside	1.84 ± 0.55	−0.52	Down
	Kaempferol-3-*O*-rhamnoside	1.11 ± 0.91	−0.28	Down
	Petunidin-3-*O*-galactoside	1.58 ± 0.86	0.31	Up
	Cyanidin-3-*O*-(6’’-caffeoyl-glucoside)	1.01 ± 0.50	0.34	Up
	Peonidin-3*-O*-galactoside	1.65 ± 1.04	0.35	Up
	3-Methoxysinensetin	1.01 ± 0.50	0.43	Up
	Cyanidin-3-*O*-(6’’-acetyl-galactoside)	1.11 ± 1.30	0.62	Up
	Gardenin B	2.00 ± 0.49	0.76	Up
	Chrysin	1.52 ± 1.02	1.20	Up
	Daidzin	1.16 ± 1.23	1.66	Up
	Apigenin-6,8-*C*-galactoside-*C*-arabinoside	1.35 ± 0.79	2.51	Up
Lignans	Lariciresinol	1.12 ± 0.81	0.09	Up
Other polyphenols	Psoralen	1.27 ± 1.11	−1.07	Down
	Coumarin	1.18 ± 0.48	0.35	Up
	*p*-Anisaldehyde	1.26 ± 0.72	0.36	Up
Phenolic acids	3,4/3,5-Diferuloylquinic acid	1.14 ± 1.28	−13.62	Down
	Ferulic acid	1.27 ± 1.02	−1.64	Down
	2,4/2,6-Dihydroxybenzoic acid; Gentisic acid; Gallic aldehyde	1.03 ± 1.15	0.26	Up
	*p*-Coumaric acid ethyl ester	1.09 ± 1.02	1.84	Up
	Sinapic acid	1.10 ± 0.59	2.02	Up
***Red Rooibos***				
Flavonoids	Xanthohumol; Isoxanthohumol	1.54 ± 1.55	−9.59	Down
	Peonidin-3-*O*-(6’’-*p*-coumaroyl-glucoside)	1.46 ± 1.58	−2.36	Down
	Gardenin B	1.50 ± 1.89	−2.16	Down
	Hemiphlorin; Naringenin-6-*C*-glucoside; Naringenin-7-*O-*glucoside	1.40 ± 1.04	−2.08	Down
	Kaempferol-7-*O*-glucoside	1.51 ± 1.55	−1.90	Down
	Chrysoeriol-7-*O*-glucoside; Isorhamnetin-3-*O*-rutinoside	1.13 ± 1.48	−1.70	Down
	Chrysoeriol; Hispidulin	2.22 ± 0.51	−1.50	Down
	Naringin	1.28 ± 1.71	−1.42	Down
	Kaempferol-3-*O*-(6’’-acetyl-galactoside)-7-*O*-rhamnoside	1.53 ± 1.01	−1.39	Down
	Peonidin	1.61 ± 1.21	−1.36	Down
	Phloretin-2’-*O*-xylosyl-glucoside	1.06 ± 0.86	−1.09	Down
	Pelargonidin-3-*O*-sambubioside	1.15 ± 0.66	−1.07	Down
	Petunidin-3-*O*-(6’’-acetyl-galactoside)	1.15 ± 0.77	−0.18	Down
	Chrysin	1.23 ± 1.49	0.79	Up
	Malvidin-3-*O*-glucoside	1.51 ± 0.74	1.12	Up
	6-Geranylnaringenin	1.13 ± 1.15	1.89	Up
	Kaempferol-3-*O*-acetyl-glucoside	1.04 ± 0.95	1.96	Up
	3-Methoxysinensetin	1.31 ± 0.78	2.24	Up
	Peonidin-3-*O*-galactoside	1.16 ± 0.77	2.53	Up
	Isorhamnetin-3-*O*-galactoside	1.39 ± 0.92	5.73	Up
	Cyanidin-3-*O*-(6’’-acetyl-galactoside)	1.03 ± 1.14	6.38	Up
Lignans	Lariciresinol	1.11 ± 1.23	1.02	Up
Other polyphenols	Psoralen	1.73 ± 1.16	−10.28	Down
	Juglone	1.25 ± 1.15	−4.46	Down
	3,4-DHPEA-EDA	1.53 ± 1.13	−1.79	Down
	Ligstroside	1.07 ± 1.69	0.33	Up
	5-Pentadecylresorcinol	2.44 ± 0.25	1.33	Up
	*p*-Anisaldehyde	1.23 ± 1.09	1.74	Up
	4-Hydroxycoumarin	1.13 ± 0.71	2.40	Up
Phenolic acids	Feruloyl glucose	1.03 ± 0.38	0.11	Up
	Ferulic acid	1.15 ± 0.83	0.39	Up
	Sinapine	1.22 ± 0.80	1.93	Up
	4-Hydroxybenzoic acid	1.33 ± 0.79	10.91	Up
Stilbenes	Piceatannol-3-*O*-glucoside	1.43 ± 0.76	1.49	Up

## Supplementary Materials

The following are available online at https://www.mdpi.com/2076-3921/8/10/499/s1, Table S1: Dataset containing all polyphenols putatively annotated in each sample, together with abundances, composite spectrum and MS-MS spectra of compounds confirmed via tandem mass spectrometry. ABTS, ORAC, TPC, Aspalathin values, together with VIP markers of the comparison green vs red and Pearson’s correlation coefficients are also provided.
